# Prediction of width of un-erupted incisors, canines and premolars in a Ugandan population: A cross sectional study

**DOI:** 10.1186/1472-6831-12-23

**Published:** 2012-07-23

**Authors:** William Buwembo, Annet Kutesa, Louis Muwazi, Charles Mugisha Rwenyonyi

**Affiliations:** 1 Department of Anatomy, College of Health Sciences, Makerere University, Kampala, Uganda; 2 Department of Dentistry, College of Health Sciences, Makerere University, Kampala, Uganda

**Keywords:** Canines, Incisors, Mesio-distal widths, Prediction equation, Premolars, Ugandan

## Abstract

**Background:**

Accurate prediction of the space forms an important part of an orthodontic assessment in the mixed dentition. However the most commonly used methods of space analysis are based on data developed on Caucasian populations. In order to provide more accurate local data we set out to develop a formula for predicting the widths of un-erupted canines and premolars for a Ugandan population and to compare the predicted widths of the teeth from this formula with those obtained from Moyers’ tables, and Tanaka and Johnston’s equations.

**Methods:**

Dental casts were prepared using mandibular and maxillary arch impressions of 220 children (85 boys/135 girls) aged 12–17 years recruited from schools in Kampala, Uganda. The mesio-distal width of the mandibular incisors, mandibular and maxillary canines and premolars were measured with a pair of digital calipers. Based on regression analysis, predictive equations were derived and the findings were compared with those presented in Moyers’ probability tables, and Tanaka and Johnston’s equations.

**Results:**

There were no statistically significant differences between the tooth widths predicted by our equations and those from Moyers’ probability tables at the 65^th^ and 75^th^ percentile probabilities for the girls and at 75^th^ level in boys in the mandibular arch. While in the maxillary arch no statistically significant differences at the 75^th^ and 95^th^ levels were noted in girls. There were statistically significant differences between predicted tooth sizes using equations from the present study and those predicted from the Tanaka and Johnston regression equations.

**Conclusions:**

In this Ugandan population, Moyers’ probability tables could be used to predict tooth widths at specific percentile probabilities, but generally, Tanaka and Johnston technique tends to overestimate the tooth widths.

## Background

Accurate prediction of the space available to accommodate the un-erupted canines and premolars forms an important part of an orthodontic assessment in the mixed dentition [1-4] as it is reported to assist dental practitioners to determine the treatment options for the patients [2]. Different methods have been employed to predict space for un-erupted teeth [3-17]. The most commonly used methods are Moyers’ probability tables [5] and the prediction equation of Tanaka and Johnston [14]. However, these methods were developed on Caucasian populations and their predictive accuracy on populations from other races is doubtful [17-23]. Consequently, this led to development of prediction equations and probability tables for different populations [19,20,24-26]. So far we have not come across any information regarding development of formulae for predicting widths of un-erupted teeth in a Ugandan population. The purpose of this study was to develop a formula for predicting the widths of un-erupted canines and premolars in a Ugandan population and to compare the predicted values with those obtained from methods developed by Moyers [5], and Tanaka and Johnston [14].

## Methods

### Study design

This was a cross sectional study based on casts of the mandibular and maxillary arches

### Study area

The study areas were 5 secondary schools within a radius of 6 km from Makerere University College of Health Sciences, the affiliate institution of the investigators. The 5 study schools were randomly selected from a list of 35 schools using numbers.

### Study population

The study sample comprised school children (n = 220) aged 12 – 17 years who had all permanent teeth erupted except third molars. All the children who were Ugandans of African descent in the above age range were requested to make two lines according to sex. They were systematically randomly selected. Every 5^th^ child was selected from the line, totaling 232 children. The children were clinically examined for dentition status and those who had crowded, spaced, malformed or missing (except third molar) teeth, inter-proximal caries, inter-proximal restorations or had history of orthodontic treatment were excluded. The children were excluded as they were assumed not to have normal contact points on proximal tooth surfaces. Twelve children were thus excluded leaving a sample of 220 children (85 boys/135 girls).

### Ethical issues

Permission to carry out the study was obtained from Makerere University Medical School Research and Ethics Committee and the respective school authorities. The purpose of the study was explained to the parents/guardians and the children in accordance with Helsinki Declaration [27]. Written consent was obtained from the parents/guardians of the children. All the children assented to the study and were recruited.

### Study dental casts

Each child’s impressions of mandibular and maxillary arches were taken using disposable dental trays (Acumen Surgical Pvt Ltd, Barcelona, Spain) and sodium alginate impression material (Kromopan Lascod Spa, Italy). The impressions for each child were wrapped in wet cotton wool and kept in a separate polythene bag with an identification number and then transported within 2 h to the dental laboratory in the Department of Dentistry, Makerere University College of Health Sciences for casting. On average, the impressions were poured into casts using dental stone (Gypsano LLC, Fujariah, United Arab Emirates) within 6 hr. The casts were let to set for 3 h before separating them from the impressions. The respective identification number was inscribed on each pair of dental casts.

### Measurement of tooth mesio-distal width

In order to provide for reliability and consistency in tooth width measurements, one of the investigators (WB) did all the measurements on the dental casts with a pair of digital sliding vernier calipers (Mitutoyo, Southampton, U.K) in natural light as described by Hixon and Oldfather [11]. The mesio-distal widths of the teeth were obtained by measuring the greatest distance between the contact points on proximal tooth surfaces [11,28]. The mesio-distal width of the four mandibular permanent incisors, maxillary and mandibular canines and premolar teeth were measured. The measured values were corrected to the nearest 0.01 mm and recorded onto a data sheet with their corresponding identification numbers.

### Reliability test

Blind duplicate measurement of the tooth widths in 10% (n = 22) of the dental casts was done to assess reproducibility at an interval of 1 week. This was based on systematic random selection of every 10^th^ cast (mandibular and maxillary) from the sample. The intraclass correlation coefficients were calculated to check for consistency [29] in the measurement of tooth widths on the 22 sets of dental casts. The agreement was almost perfect with correlation coefficients between 0.84 and 0.93 for summations of mandibular incisors and all four sets of canine and premolar widths.

### Data analysis

The data were entered into a computer and analysed using Statistical Package for Social Sciences Inc. (SPSS version 15.0 for Windows, Illinois, USA). Frequency distributions were used to describe the material and the data assumed a normal distribution. Student’s *t* test for independent samples was used to assess any significant difference: (i) between the sums of the mesiodistal diameter of permanent: (a) incisors, (b) canine and first and second premolars, canine and first and second premolars in male and female subjects, and (ii) between the regression values using Moyers’ probability tables [5] and the Tanaka and Johnston equation [14] with the actual canine and premolars width measurements. Bivariate analyses and linear regression analyses were performed between the predicted and actual tooth size for the Tanaka and Johnston prediction equation [14]. The following predictive formula was derived: Y = a + b(x), where Y was the predicted sum of widths of the canine and premolars in one quadrant; x was the measured width of the mandibular incisors while a and b were constants. The interclass correlation for parametric data was used to check for reproducibility in measuring the tooth widths. The level of significance was set at 5%. The values of the sum of the canines and premolars obtained using the derived prediction formulae were compared with the predicted values obtained with Moyers’ probability tables [5], and the Tanaka and Johnston’s prediction equation [14].

## Results

The study population comprised of 220 children aged 12–17 years (mean, 14.6 ± 3.5 years). The width of maxillary and mandibular canine and premolars was significantly higher in the boys as compared to girls (p < 0.05, *t* test; Table 1). There was a significant correlation between the mandibular incisors, and sum of the maxillary canine and premolars, and the sum of the mandibular canine and premolars (p < 0.05, *t* test; Table 2). The overall regression coefficient of mandibular canine and premolars was 0.83 and for maxillary canine and premolars was 0.78 (Table 2). The standard error of estimate ranged from 0·30 to 0·52 mm with the errors smaller in girls as compared to boys (Table 2). The values of regression coefficient b ranged from 0.54 to 0·68 and were all significantly different (p < 0·05, *t* test). Tables 3 and 4 show the differences between the sizes of canine and premolars widths obtained using the regression equations from the present study and the predicted widths of canine and premolars for mandibular and maxillary arches derived from Moyers’ probability tables [5] at different probability levels, respectively. There were no statistically significant differences between the sizes predicted by the equations and the predicted widths from Moyers’ probability tables [5] at the 65% level for the boys and 75% level for the girls in the mandibular arch (p > 0.05, *t* test; Table 3). In the maxillary arch, no significant difference between the sizes predicted by the equations and the predicted widths from Moyers’ probability tables [5] at 75^th^ level in boys and at 75^th^ and 95^th^ levels in the girls (p > 0.05%, *t* test; Table 4). The difference (mm) between the regression values derived using the equations from the present study of the sum of the canine and premolars of Ugandan children and those predicted from Tanaka and Johnston equations [14] for same subjects in the mandibular and maxillary arches is presented in Table 5. There were statistically significant differences between predicted widths using equations from the present study compared to Tanaka and Johnston regression equations [14] (p < 0.05, *t* test; Table 5).

**Table 1:** The measurement of the sum of mesiodistal width of the mandibular incisors, mandibular and maxillary canines and premolar teeth (mm) according to sex of the children

**Sex**	**Tooth group**	**Mean**	**S.D**	**Standard error of mean**
Girls (n = 135)	Mandibular incisors	20.99	2.34	0.202
	Mandibular canines and premolars	19.99	1.89	0.163
	Maxillary canines and premolars	20.53	1.71	0.140
Boys (n = 85)	Mandibular incisors	21.53	2.49	0.270
	Mandibular canines and premolars	20.62	1.94	0.210
	Maxillary canines and premolars	21.05	1.76	0.191
Total (n = 220)	Mandibular incisors	21.20	2.41	0.163
	Mandibular canines and premolars	21.24	1.93	0.130
	Maxillary canines and premolars	20.73	1.75	0.118

S.D, Standard deviation.

**Table 2:** Regression parameters for the prediction equations of the sum of widths of un-erupted mandibular and maxillary canine and premolar teeth according to sex of the children

			**Regression coefficient**				
**Sex**	**Tooth group**	**r**	**a**	**B**	**95% Confidence interval**	**SEE**	**P-value**
Girls (n = 135)	Mandibular canines and premolars	0.84	5.73	0.68	0.61-0.75	0.37	<0.001
	Maxillary canines and premolars	0.79	8.49	0.57	0.50-0.65	0.39	<0.001
Boys (n = 85)	Mandibular canines and premolars	0.79	7.34	0.62	0.51 - 0.72	0.52	<0.001
	Maxillary canines and premolars	0.77	9.32	0.54	0.45 - 0.64	0.50	<0.001
Combined (n = 220)	Mandibular canines and premolars	0.83	6.25	0.66	0.60 - 0.72	0.31	<0.001
	Maxillary canines and premolars	0.78	8.72	0.57	0.51 - 0.63	0.30	<0.001

SEE, Standard error of estimate; r, Coefficient of correlation; a and b, Linear regression constants.

**Table 3:** **The mean difference (in mm) between the predicted values of of the mandibular canines and premolar teeth from the present study and those predicted from Moyers’ tables**[5]**for the same subjects at deferent percentiles (5 – 95%)**

**Percentile probability (%)**	**Girls (n = 135)**				**Boys (n = 85)**			
	**Mean difference (mm)**	**SD**	**95% CI**	**P-value**	**Mean difference (mm)**	**SD**	**95% CI**	**P-value**
5	−2.66	0.28	−2.84 - -2.48	<0.001	2.32	0.29	2.13 - 2.51	<0.001
15	−1.93	0.28	−2.11 - -1.75	<0.001	1.61	0.30	1.42 -1.80	<0.001
25	−1.50	0.27	−1.67 - -1.32	<0.001	1.16	0.28	0.98 -1.34	<0.001
35	−1.15	0.28	−1.33 - -0.97	<0.001	0.81	0.30	0.62 - 1.01	<0.001
50	−0.70	0.29	−0.87 - -0.50	<0.001	0.37	0.	0.17 - 0.57	0.002
65	−0.22	0.29	−0.40 - -0.02	0.027	−0.08	0.32	−0.28 – 0.12	0.388
75	0.13	0.31	−0.07 - 0.32	0.195	−0.43	0.34	−0.65 - -0.21	0.001
85	0.55	0.30	0.35 - 0.74	<0.001	−0.86	0.33	−1.06 - -0.64	<0.001
95	1.28	0.31	1.08 - 1.48	<0.001	−1.60	0.36	−1.84 - -1.37	<0.001

CI, Confidence interval; SD, Standard deviation.

**Table 4:** **The mean difference (in mm) between the predicted values of the sum of maxillary canines and premolar teeth of the present study and those predicted from Moyers’ tables**[5]**for the same subjects at deferent percentiles (5 – 95%)**

**Percentile probability %**	**Girls (n = 135)**				**Boys (n = 85)**			
	**Mean difference (mm)**	**SD**	**95% CI**	**P value**	**Mean difference (mm)**	**SD**	**95% CI**	**P value**
5	−2.47	0.47	−2.77 - -2.17	<0.001	−1.61	0.08	−1.66 - -1.56	<0.001
15	−1.81	0.48	−2.12 - -1.51	<0.001	−1.09	0.07	−1.14 - -1.04	<0.001
25	−1.44	0.49	−1.75 - -1.12	<0.001	−0.79	0.07	−0.84 - -0.74	<0.001
35	−1.14	0.49	−1.45 - -0.82	<0.001	−0.54	0.09	−0.59 - -0.48	<0.001
50	−0.74	0.49	−1.05 - -0.42	<0.001	−0.20	0.06	−0.25 - -0.16	<0.001
65	−0.33	0.50	−0.65 - -0.01	0.044	0.11	0.08	0.06 - 0.16	0.001
75	−0.02	0.49	−0.34 - 0.29	0.865	−1.30	5.73	−4.96 - 2.36	0.451
85	0.34	0.51	0.01 - 0.66	0.041	0.67	0.10	0.60 - 0.74	<0.001
95	−0.67	5.94	−4.45 - 3.10	0.702	1.20	0.10	1.14 - 1.27	<0.001

CI, Confidence interval; SD, Standard deviation.

**Table 5:** **The mean difference (in mm) between the predicted values of the sum of the permanent canines and the first and second premolars (345) of Ugandan subjects and those predicted from Tanaka and Johnston equation (0.5 Sum of lower incisor widths plus 11 for the upper 345 or 0.5 the sum of the lower incisor widths plus 10.5 for the lower 345)**[14]**for the same subjects**

**Sex**	**Arch**	**Mean difference**	**Standard deviation**	**95% Confidence interval of the difference**
Total	Maxillary canine and premolars	0.75*	0.11	0.66 - 0.81
	Mandibular canine and premolars	−0.70*	0.25	−0.86 - -0.53
Boys	Maxillary canine and premolars	−0.85*	0.10	−0.91 - -0.78
	Mandibular canine and premolars	−0.53*	0.22	−0.67 - -0.38
Girls	Maxillary canine and premolars	−0.98*	0.12	−1.06 - -0.90
	Mandibular canine and premolars	−0.80*	0.33	−1.02 - -0.59

*P-value <0.05.

## Discussion

Prediction of the width of un-erupted teeth in a population based on values from another race is likely to provide inaccurate estimates [18,19,23,24]. This may be attributed to racial differences in tooth sizes [25]. Data to provide such equations for a Ugandan population for the first time were generated in the present study. The correlation coefficients between the width of the mandibular incisors, mandibular and maxillary canines and premolar teeth ranged from 0.77 to 0.84 (Table 2) using the derived prediction formulae. Our correlation coefficients were higher compared to those from Hong Kong Chinese (r = 0.58 to 0.66) [19], Jordanian (r = .60 to 0.66) [28], Syrian (r = 0.58 to 0.66) [26] and the Pakistan population (r = .64 to 0.67) [30]. Comparable to our findings, a study from a Brazilian population [31] found a correlation value of 0.81. The differences in correlation values may be due to the genetic influence on the tooth sizes [25,30]. We also found a sex difference in the correlation values which may partly be attributed to the size of the canines and premolars in the maxillary and mandibular arches (Table 1) as previously reported [30].

In the present study, the slope of the simple linear regression equation ranged from 0·54 for the maxillary teeth in the boys to 0·68 for the mandibular teeth in the girls (Table 2). These values are comparable to those reported for the African American [17,32], Thai [33], Hong Kong Chinese [19], Senegalese [34], Saudi [18] and Jordanian populations [28].

Moyers [5] recommended using the 75th percentile level of probability in his tables to predict mesio-distal widths of un-erupted permanent teeth, however, previous studies showed that Moyers’ probability tables are not an accurate method for the prediction of mesio-distal widths of un-erupted permanent teeth in different populations [18-28,30-36].

In the present study there were no statistically significant differences between the sum of the sizes of the canines and premolars predicted by our equations and the predicted widths from Moyers’ tables [4] at the 65^th^ and 75^th^ percentile levels of probability in boys and girls, respectively, in the mandibular arch (Table 3). Furthermore, we observed no statistical significant differences between the sum of the sizes of the maxillary canine and premolars predicted from our equation and the predicted width from Moyers’ tables [5] at 75^th^ percentile level of probability in boys as well as 75^th^ and 95^th^ levels in girls (Table 4). This is an indication that Moyers’ probability tables can be used to predict un-erupted tooth widths in a Ugandan population at those particular percentile levels of probability. In a previous study in the Jordanian population [28], it was found that Moyers’ probability tables [5] for prediction of sizes of un-erupted permanent teeth can be applied at 65^th^ and 75^th^ levels in male and at the 85^th^ level in female subjects for both maxillary and mandibular arches. However, in the Saudi Arabian population [24], the most accurate width of un-erupted permanent canine and premolars was predicted at 50^th^ percentile level of probability as compared to the most commonly used 75^th^ level when both sexes are combined. These variations in percentile levels of probability used in predicting the most accurate tooth widths call for the need to develop regression equations for the different populations.

It is evident from the findings of the present study (Table 5) that the Tanaka and Johnston technique [14] overestimates the actual size of the Ugandan tooth widths. This overestimation can partly be explained by racial differences between the present study and that of Tanaka and Johnston [14]. However, our finding is in support of some previous workers [18,19,33] who also reported an overestimation of the size of un-erupted canines and premolars when using the Tanaka and Johnston prediction equations [14]. On the other hand, other workers [28] reported an underestimation of the actual mesio-distal widths of un-erupted permanent teeth when using the same technique. It can therefore be concluded that because of the differences in mesio-distal widths of mandibular incisors, mandibular and maxillary canines and premolars among different racial groups, data collected from one ethnic group for the purpose of predicting the size of un-erupted permanent teeth might generally, not be applicable to another [18].

## Conclusions

Moyers’ probability tables could accurately be used to predict tooth widths of Ugandan population at the 65^th^ and 75^th^ percentile levels of probability in boys and girls, respectively, in the mandibular arch, and at 75^th^ level in the boys as well as 75^th^ and 95^th^ levels in girls in the maxillary arch. However, the Tanaka and Johnston prediction equation overestimates the actual size of tooth widths of the Ugandan population.

## Competing interests

The authors declare that they have no competing interests.

## Authors’ contributions

WB participated in measurement of tooth widths on dental casts and manuscript writing. AK and ML participated in data analysis and manuscript writing. CMR conceived the idea of study and participated in its design and coordination. All authors read and approved the final manuscript.

## Pre-publication history

The pre-publication history for this paper can be accessed here:

http://www.biomedcentral.com/1472-6831/12/23/prepub
